# Dietary Modifications in Critically-Ill Patients: A Comparison of Persian Medicine and Conventional Medicine Perspectives

**DOI:** 10.1155/2023/5069471

**Published:** 2023-01-05

**Authors:** Mohammad Ali Zareian, Mahdi Alizadeh Vaghasloo, Narges Sharifi Darani, Mohammad Ansaripour, Alireza Asghari, Laila Shirbeigi, Fatemeh Nejatbakhsh

**Affiliations:** ^1^Department of Traditional Medicine, School of Persian Medicine, Tehran University of Medical Sciences, Tehran, Iran; ^2^Persian Medicine Network (PMN), Universal Scientific Education and Research Network (USERN), Tehran, Iran; ^3^Canadian Association of Persian Medicine, Montreal, Canada; ^4^Department of Persian Medicine, Faculty of Medicine, Isfahan University of Medical Sciences, Isfahan, Iran; ^5^Nutrition and Food Security Research Center, Isfahan University of Medical Sciences, Isfahan, Iran; ^6^Association Québécoise Des Thérapeutes Naturels, Montreal, Québec, Canada; ^7^Canadian College of Integrative Medicine, Toronto, Canada; ^8^Food Microbiology Research Center, Tehran University of Medical Sciences, Tehran, Iran

## Abstract

In Persian Medicine (PM) literature, a crisis is the culmination of the body's response to illness, which necessitates fundamental dietary modification to improve prognosis. In this narrative review, authentic PM textbooks as well as articles on diets for critically-ill patients (CIPs) obtained from PubMed and Google Scholar databases, were reviewed, and after gathering data, they were classified, coded, analyzed, and compared. In the acute phase, both PM and conventional medicine agree on relative food restriction, but PM lays a special focus on the use of meat in cases of weakness. There are both similarities and differences between PM and conventional medicine regarding nutritional recommendations in critical illness. For example, recommendations for food restriction and protein intake are similar in both schools, but recommendations for carbohydrate intake are different. The variables addressed and emphasized in PM require further evaluation in clinical trials.

## 1. Introduction

Critically-ill patients are those who require continuous monitoring and artificial support of more than one vital organ [1]. Malnutrition in critically-ill patients is directly related to infectious complications and length of hospitalization, and providing proper nutrition for these patients is part of the standard treatment of these patients [2, 3]. If the patient tolerates it, enteral nutrition is the preferred route for nutritional support of critically-ill patients under special care [2, 4, 5]. These patients usually require more nutrients and energy as a result of catabolic stress. In order to meet the nutritional needs of patients admitted to the intensive care unit, it is necessary to create a diet plan using oral nutritional compounds as soon as tolerated, because nonenteral nutrition has adverse gastrointestinal and other complications [4]. In addition to preserving the mucosa of the digestive tract and strengthening the functions of its neuroendocrine system, enteral nutrition reduces complications (less infection rate, better wound healing, reduces the duration of mechanical ventilation, reduces the length of stay in the ICU and hospital, and accelerates recovery), and reduces mortality. In the absence of anatomic bowel discontinuity or splanchnic ischemia, early enteral feeding within 24 to 48 hours of ICU admission is beneficial and supported by some evidence [1].

One of the oldest medical schools dating back thousands of years, traditional Persian Medicine (PM) has consistently considered an appropriate diet to be the first step in treatment. Rhazes (10th century), a well-known Persian philosopher and physician, paid special attention to the importance of diet therapy in treating patients. In his legendary phrase, “Do not use medicine until you can treat patients with food,” he has advised physicians to put this principle into practice [6]. PM physicians used several categories such as absolute aliment, functional food, and pharmaconutrient to define and classify foods, which demonstrates their concern for determining the qualities and uses of each food item in various health contexts [7, 8]. Dietary guidelines are an important aspect of the PM treatment strategy for most diseases, including crises and acute illnesses such as fever, as well as critically-ill patients (CIPs) [9, 10].

The critical stage of a disease is one of the medical challenges and acute conditions, in which there is a serious need to modify the diet. According to PM sources, a crisis is a period of severe and sudden symptoms, particularly in acute diseases. This stage can culminate in a variety of outcomes, including full recovery, chronic illness, or death, depending on individual and environmental factors. At this point, the physician must constantly monitor signs and symptoms to forecast disease progression and take necessary action [11–13].

During a crisis, some fluid is expelled from the body via perspiration, bleeding, diarrhea, urination, and vomiting. Persian scholars believed that the body normally picks the best feasible way for fluid excretion to maintain fluid balance and that the physician should not block or slow down this path initially but rather facilitate the process with therapeutic measures if required [14]. Shortly before the crisis, the body modifies fluid consistency and concentration to facilitate excretion. In PM literature, this modification is referred to as “Nozj,” which is one of the fundamental concepts of PM [15].

PM scholars deemed completion of the Nozj process before a crisis as important in order for the patient to fully recover from the crisis. Indeed, severe symptoms, such as high fever and neurological symptoms, frequently ameliorate after *Nozj* and total excretion of morbid materials, thereby improving the health condition. Accordingly, therapeutic measures should firstly not prohibit Nozj, and secondly, facilitate it. During a crisis, it is also critical that the fluid is excreted via the appropriate route. All these situations need constant medical supervision; as improper intervention may worsen the prognosis [12, 16].

Since the crisis is the culmination of the body's fight against the disease and necessitates concentration of all body forces to overcome and defeat the disease, consuming heavy and slow-digesting food that affects body forces, including digestion, can be a mistake and even deadly. Temporary food prohibition may be required in some instances [17]. PM nutrition concepts and recommendations seem to be useful in the management and need to be reviewed. Hence, this article endeavors to compare the perspectives of PM and modern medicine on diet control in CIPs.

## 2. Materials and Methods

In this study, which is a content analysis type, the theoretical sampling method was followed. Theoretical sampling is a special type of criterion-based sampling that follows the gradual selection rule. In this method, the researcher takes a primary source, analyses the data, and then retakes more samples to refine his emerging categories and theories. This process continues until the researcher reaches the stage of data saturation; that is, the stage where no new insights and ideas are obtained from further expansion of examples [18]. In this study, “Canon of Medicine” of Avicenna (980–1032 CE) was chosen as the primary source for data extraction, and other sources including “Al-Murshid” and “Al-Hawi” of Rhazes (865–925 CE), “Kamil al-Sina'a” of Haly Abbas (949–982 CE), “Kholasah al-Hikmah” of Aghili (18th century), “Exir-e Azam” of Hakim Mohammad Azam Khan Chishti (1814–1902 CE), and “Zakhire Kharazmshahi” of Jorjani (1041–1136 CE) were used to refine and complete the data. We initially searched PM references, for CIP-related conditions including “crisis.” Subsequently, we gathered data on associated dietary modifications. In the next step, we searched PubMed and Scopus databases using the term “critically-ill patients” along with “diet” or “nutrition.” After collecting the data, open coding, and then central coding (phenomenon, causes, contexts, contexts, intervening conditions, strategies, and consequences) were done. Finally, obtained results underwent content analysis.

## 3. Results and Discussion

### 3.1. Dietary Modifications in PM

**Figure 1:**
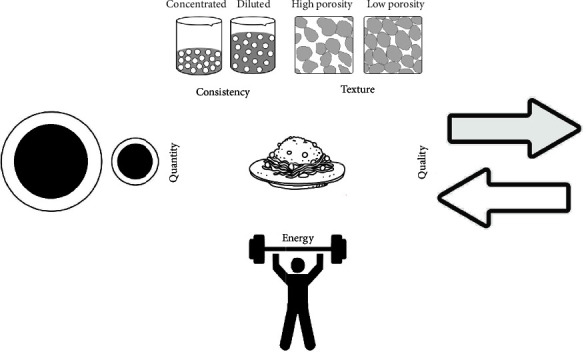
Factors to consider in improving diet in critically-ill patients.

The consistency and texture, amount (quantity), quality, and nutrition of foods are the most significant features to adjust the diet for CIPs in a crisis period (Figure 1). In general, these considerations are vital in acute diseases because of the malaise and rapid disease progression.

#### 3.1.1. Consistency and Texture of Food

PM categorizes foods into dilute or concentrated in terms of consistency, and tenuous or thick (low and high porosity) in terms of texture. Tenuous and dilute meals are similar to the clear liquid diet, liquid diet, soft diet, and semisolid diet, which are described in modern nutrition textbooks. According to PM, these foods, are more easily digested and assimilated by the body [16, 17]. In severe diseases, the diet should be more tenuous and dilute than the regularly-consumed diet, as long as there are no signs of weakness [17], because, in general, thicker and more concentrated foods are more nutritive value [16]. Complete food deprivation is the utmost approach in food restriction strategy and is advised if the appropriate conditions are met. In cases where food deprivation is not feasible, very dilute honey water and very dilute sugar water are ideal foods for the patient [17]. More details are provided in Table 1.

#### 3.1.2. Amount of Food

There are two methods to modify the quantity of food: changing the volume of each meal or changing the number of meals. Appetite and digestive status, which are directly connected to the amount of ingested food, are the most essential elements in determining the amount of food consumed [16, 17, 19]. When there is a need to increase the concentration of food to maintain strength, food should be consumed in small amounts and more frequent meals so that the digestive system is not overburdened and body powers are not diverted from fighting the disease [17].

#### 3.1.3. Food Quality

The general guideline on selecting the quality of food is that it should be the opposite of disease quality, both in terms of primary qualities (e.g., hotness, coldness, wetness, or dryness) and secondary characteristics (e.g., laxation and diuresis), like using hot antidiarrheal food in cold diseases accompanied by severe diarrhea [17, 20].

#### 3.1.4. Nutritive Value of Food

Nutritive value is the quantity of heat and moisture that food provides for the body, which is similar to energy and nutritional substances [21]. The quantity of food reserves accessible to the body is the most essential factor in determining the nutritive value of food. When levels of body reserves are high, foods with low nutritive value are used. In contrast, low reserves necessitate the use of foods with high nutritive value [17, 19].

### 3.2. Principles of Diet Modification in PM

According to PM sources, the most essential factors in selecting appropriate foods for patients include disease duration, disease stage, disease quality, patient strength or weakness, digestive strength, appetite, body reserve status, eating habits, and body mass [17, 19].

#### 3.2.1. Disease Duration

In PM, diseases are classified as acute or chronic based on their duration. Acute disease refers to diseases that last less than 40 days. A more detailed classification is presented in Table 1 [16, 17]. In “Kitab Kamil as-Sina'aaṭ-Ṭibbiyya,” Haly Abbas (949–982 CE) has noted: “Determining whether a disease is acute or chronic is necessary for two reasons: one is to predict the prognosis of the disease and the other is to estimate the nutritional needs of the patient.” He has also discussed that due to the existence of a crisis stage in acute diseases and due to the rapid progression of stages in such diseases, light foods are needed so that the body can focus on changing the concentration of disease-causing substances in order to eliminate them (a process called Nozj or coction) instead of focusing on digesting food [22].

PM physicians reason that “although food boosts strength and increases the power against sickness, it also may strengthen the pathogen.” Because of the short duration of acute diseases, the body can use existing food stores without getting weak. Therefore, in such diseases, tenuous and dilute foods should be used or eating should be completely prohibited in certain situations [21].

As a result, the shorter the illness, the lighter the meals and more tenuous and dilute foods, and the longer the illness, the heavier the meals and thicker and more concentrated foods should be [16, 17, 19]. Table 1 shows the categories of acute diseases depending on their duration, as well as examples of meals that are appropriate for each.

#### 3.2.2. Disease Stage

Regardless of duration, PM sources consider four stages for illnesses: onset, ascend, plateau, and descend. The beginning of symptoms is the onset stage. The second stage, or ascend, is when the disease condition aggravates. When symptoms reach a peak and are somewhat stabilized, the disease is in the plateau stage. At this point in time, a crisis frequently develops as it is the conclusion of a fight between body powers and sickness. The fourth or final stage is referred to as descend because the symptoms consistently ameliorate and the body powers overcome the illness. Diseases that lead to death do not have a descend stage. Aside from the fact that nutritional demands change in each of these stages, dietary interventions can also have an impact on each of these stages [16, 17, 21].

#### 3.2.3. Disease Quality

As denoted previously, the quality of food (hotness, coldness, wetness, and dryness) should be opposite the quality of disease [17, 21].

#### 3.2.4. Body Strength

If the patient is weak, even in diseases or stages where light foods should be used or food should be forbidden, the physician has to prescribe heavier meals and thicker foods to strengthen the patient and prepare him to stand against the disease [16, 17, 19].

#### 3.2.5. Digestive Power

When a patient needs heavy meals and thick foods but is unable to digest them due to poor digestion, tenuous foods must be used until the poor digestion is resolved [16, 17, 19]. According to PM, the following symptoms suggest poor digestion and thus, the necessity to reduce the density or volume of food [22, 24]:

- Feeling of heaviness in the stomach
- Flatulence
- Nausea
- Frequent burping
- Unpleasant and sour tastes in the mouth
- Hiccups after eating
- Feeling of tightness in the muscles after eating
- Early or delayed passage of food through the stomach
- Poor quality of sleep causing subsequent drowsiness after waking up
- Puffiness under the eyes
- Feeling of heaviness in the head
- Disturbances in defecation

#### 3.2.6. Patient's Appetite

Paying attention to the patient's appetite is important in two aspects. On the one hand, if the patient craves foods that are not suitable, they should be given a small amount of that food along with useful foods [25]. On the other hand, if the patient has a strong appetite for food, the amount of food should be increased to satisfy their desire and prevent them from weakening [16, 17].

#### 3.2.7. Status of Body Reserves

In the case of lacking food reserves, the body needs nutrients, and therefore both the quantity of food and its nutritive value should be increased. The volume and nutritive value of food should be reduced in case of high body reserves [16].

#### 3.2.8. Eating Habits

Patients that have been eating a lot during health, should gradually lower food volume and avoid withholding food all at once. However, for those who priorly consumed less amounts of food, the amount of food may be swiftly reduced, and the situation may be managed with more tenuous foods [17].

#### 3.2.9. Body Mass

In terms of body mass, PM categorizes individuals as lean or obese, muscular or nonmuscular, and, in terms of body tissue porous or condensed. Patients with porous bodies have more open pores and more imperceptible decay. Individuals with this type of body should not be restricted from eating during sickness, and the patient should be fed in accordance with the disease conditions. Condense bodies, on the other hand, have less decay and more closed pores and should be limited or fully prohibited from food during illness [26, 27].

**Figure 2:**
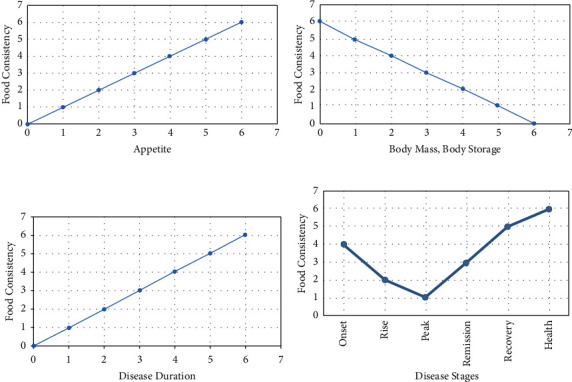
Relationships between food consistency and variables affecting diet modification. (a) The relationship between food consistency and appetite. There is a direct relationship between this variable and food consistency, i.e., the higher the appetite, the greater should be the consistency of food. (b) There is an inverse relationship between food consistency and body mass and body reserves, meaning that the higher the body mass and body reserves, the lower the food consistency should be. (c) There is a direct relationship between food consistency and disease duration; i.e., the more chronic the disease, the thicker the food should be. (d) The relationship between food consistency and disease stages is such that the concentration of food should decrease with progression from the beginning to the peak, and increase from the peak to recovery and health.

In addition to the abovementioned points, meal plans are adjusted for each disease based on the nature of the disease and specific factors that are also considered in personalized medicine. Such factors include but are not limited to age, gender, temperament, employment, previous health history, pregnancy, breastfeeding, family history, mental health, season, and climate [17]. The abovementioned issues regarding the relationship between food consistency and variables affecting diet modification are summarized in Figure 2.

### 3.3. Comparison of PM and Conventional Medicine about Nutrition in CIPs

In conventional medicine, a CIP is described as a patient who requires constant monitoring and artificial support of more than one vital organ [1]. Traditional physicians paid attention to the signs and symptoms of CIPs for constant monitoring, including the sudden and severe appearance of a crisis stage. These manifestations indicated a critical condition and the fact that the patient was in the dilemma of life and death. Some of the severe symptoms of crisis, especially neurological symptoms such as headache, delirium, and loss of consciousness, may be seen in patients labeled as critically ill by conventional medicine [13]. The similarities and differences in nutritional recommendations for CIPs in the sources of Persian and conventional medicine are summarized in Table 2.

Critically-ill patients typically require high levels of nutrients and energy due to catabolic stress. Nonintestinal feeding might have negative side effects such as disruption of intestinal microbiota and diarrhea [4]. Thus, an oral diet plan must be established to address the nutritional demands of patients as soon as they are able to tolerate food intake. In addition to maintaining the function of the intestinal mucosal barrier and the positive effect on the intestinal immune function and neuroendocrine system, oral feeding accelerates healing and reduces complications including infection, delayed wound healing, mechanical ventilation, ICU and hospital stay, and mortality. There is sufficient evidence to suggest early intestinal feeding within 24 to 48 h of ICU admission, barring any obstacle such as splanchnic ischemia or anatomical discontinuity of the intestines. For this reason, total intravenous feeding may be delayed for up to seven days even if intestinal nutrition fails, provided that the patient tolerates food intake and does not have malnutrition [1]. In PM, changes in food consistency and concentration are considered necessary in most patients to maintain intestinal nutrition, while in modern medicine, changes in food consistency are only necessary for conditions such as acute infections, gastrointestinal disorders, difficulty chewing, and following surgery [33, 34]. According to Galen, as quoted in the book “Exir-e Azam,” appropriate modification of the consistency and concentration of food in illnesses either eliminates or minimizes the need for long-term use of medications [17].

Acute stress response due to trauma or surgery, sepsis, burns, or other serious illnesses (such as myocardial infarction) leads to the secretion of cytokines, lymphokines, and hormones such as cortisol, catecholamines, and glucagon, resulting in changes in nutrient intake. These mediators counteract insulin function in the liver and adipose tissue thereby leading to insulin resistance and hyperglycemia, peripheral lipolysis, and increased hepatic gluconeogenesis and glycogenolysis. Reducing glucose oxidation, despite increasing insulin concentrations, makes limited carbohydrate stores available to vital glucose-dependent organs by preventing the use of glucose in muscle and adipose tissue. Lipid oxidation increases despite a decrease in glucose oxidation. Protein stores in muscles and organs are also broken down and used to make more essential proteins or released from peripheral tissues and transported to the liver, where they are deaminated to form glucose. The synthesis of new proteins also decreases as energy production from fat metabolism increases. For this reason, it is recommended to reduce carbohydrate intake in the acute phase [1]. Although PM authors agree on food restriction in the acute phase, they recommend the use of diluted and watery carbohydrate sources in the acute phase and for CIPs (shown in Table 1). Due to the rapid digestion of these substances and the low energy needed for digestion, they proposed this strategy as they believed that the body would have more energy to fight disease by using less energy for digestion [17]. However, recent studies have indicated that consuming large amounts of carbohydrates, either orally or intravenously, may have negative effects on CIPs, particularly when a respiratory condition is present [38]. Maintaining blood glucose levels below 120–150 mg/dL decreases mortality, shortens hospital stays, and lowers the incidence of kidney failure and blood transfusions, but consuming a balanced quantity of carbohydrates is necessary for protein production and prevention of lipolysis [1].

High calorie and protein intake during the first week, worsens prognosis in CIPs, whereas relative food restriction improves outcomes. Considering the absence of a specific standard for the extent of food restriction, this strategy is successful when the patient consumes adequate protein [1, 3, 29, 30]. There are two additional factors to take into account regarding protein supply. First, no matter how weak the patient is, the body provides some of the protein needed in itself. Certain studies have shown that patients on a high-protein diet, experience more muscle loss. Second, when a patient is not septic and is not receiving excessive calories, increasing dietary protein is advantageous for the patient. Without taking into account other considerations, increasing protein intake does not decrease mortality in CIPs [1, 39]. Meat is one of the main sources of protein in PM [16, 17]. Recent research has demonstrated the importance of mitochondrial dysfunction in CIPs and the necessity of micronutrients, such as carnitine and phosphate, for healthy mitochondrial activity and energy synthesis. Meat and animal protein sources are the most important sources of carnitine and phosphate [40, 41]. Therefore, the emphasis of PM resources on adding meat to meal plans for weak patients seems rational.

As mentioned above, relative food restriction in the early days makes sense, because it maintains the process of autophagy to recycle intracellular nutrients and maintain energy homeostasis during nutrient deprivation, enhances the immune response, and eliminates toxic masses of proteins and damaged organs. Moreover, the prevention of organ failure is essential. Invasive nutrient supply, especially via the intravenous route, can exacerbate inflammatory response by increasing immune system dysfunction and reducing resistance to infections [1]. According to PM resources, food restriction should be planned with particular regard to the patient's strength and the stage of the disease because, as shown in Figure 2(d), the process of food administration has a sinusoidal process [16]. Investigating this method in future research may reveal hidden aspects of nutrition in CIPs.

## 4. Conclusions

Considering all the issues raised in this article, it appears that both medical schools (PM and conventional medicine) accept food restrictions for CIPs who are not malnourished and weak. However, there are variations in dietary specifications. For example, PM scholars believe meat to be the most important source of diet protein, and that food restriction should be implemented according to the sinusoidal process of the patient's condition. Further clinical research should be conducted to investigate PM's recommendations for modifying the consistency and concentration of food in patients. Existing controversies in the nutritional therapy of CIPs may be resolved by paying concurrent attention to the perspectives of both schools and the accuracy of the physiopathology identified in CIPs.

## Figures and Tables

**Table 1:** Types of acute illnesses and sample foods suitable for each.

Type of illness	Period of illness	Type of suitable food	Examples
Extremely acute	4 days or less	Extremely light foods (similar to a clear liquid diet)	Highly diluted nonalcoholic barley water, very thin soup, highly diluted pomegranate juice, and highly diluted oxymel [17, 21, 23]
Very acute	Between 4–7 days	Very light foods (similar to the liquid diet)	Diluted oxymel, diluted pomegranate juice, diluted pumpkin juice, and diluted cucumber juice [17]
Acute	Between 7–14 days	Light foods (similar to the soft and semisolid diet)	Porridge, soup, thick barley water, soft-boiled egg yolk, and very small fish [17, 23]
Subacute	Between 14–27 days	Medium-light foods (similar to the semisolid and solid diet)	Chicken, lamb and goat meat, and fresh fish [17, 23]
Chronified acute	Between 27–40 days	Dense foods (similar to the solid diet)	Regular diet suitable for patients [17]

**Table 2:** Comparison of Persian medicine and conventional medicine on the nutrition of critically-ill patients (CIPs).

Topic	Conventional Medicine	Persian Medicine
Definition of a CIP	Severe conditions that require continuous monitoring and artificial support of more than one vital organ [1]	Patients in need of constant medical attention due to being in the crisis period of a disease. The body's struggle against disease has reached a peak [17]

Possible side effects in CIPs	Critical illness is usually associated with catabolic stress, systemic inflammatory response, and complications including increased incidence of infections, multi-organ dysfunction, prolonged hospital stay, and increased mortality [1, 4]. Neurological complications, such as delirium, acute ischemic stroke, intra-cerebral hemorrhage, hypoxic-ischemic brain injury, flaccid paralysis, and rhabdomyolysis, are also expected in these patients [28]	Crisis is a sudden and drastic change in the course of a disease. Depending on various factors, it ultimately leads to complete recovery, chronic illness, or death. A range of symptoms, including headache, physical and mental restlessness, delirium, and loss of consciousness, can be expected in such condition [11, 12]

Importance of proper nutrition in CIPs	Malnutrition in CIPs is directly related to infectious complications and prolonged length of hospital stay. Providing proper nutrition for these patients is part of standard treatment [2, 3]	Lack of proper nutrition in CIPs can lead to Patient deterioration, disease exacerbation, disease prolongation, increased morbidity and mortality [17]

Food restriction	Treatment outcomes are improved by relative calorie restriction in the early days, while consuming enough protein [1, 3, 29, 30]	Relative food restriction and sometimes transitory fasting is necessary in CIPs. Consuming excessive or thick and slow-digesting food can be fatal. In weak patients, receiving enough meat and food is especially important [17]

The rationale behind food restriction	Due to metabolic changes caused by secretion of hormones and cytokines, increased catabolism and hypermetabolism, food restriction seems reasonable and has positive effects on prevention and control of inflammation [1]	Regular diet, in addition to strengthening the patient, may intensify the disease [21]. On the other hand, excessive food consumption, reduces the body's ability to fight disease due to the involvement of body forces in digestion and absorption processes [17, 19]

Factors determining the amount of required energy (which is related to the amount and type of required food)	There are many determinants of energy expenditure, including the severity of trauma, sepsis, fever, age, physical activity, medications, and the duration and developmental stage of critical illness. These factors overlap in very complex ways, adding or subtracting effects of each other [1, 31]	According to PM principles, the most important variables determining the type of diet suitable for each patient include disease duration, disease stage, disease quality, patient's energy, digestive power, appetite, the status of body reserves, prior eating habits, and body mass [17, 19]

Duration of food restriction	Normally, relative food restriction is considered in the acute phase for 2-4 days. This is longer in overweight cases and shorter in case of malnutrition. When discontinuing intestinal feeding, based on patient tolerance and malnutrition, complete intravenous feeding may be delayed for up to 7 days [1]	In Al-Hawi, Rhazes allows the most severe state of food restriction between 4 to 7 days depending on physical condition of the patient [32]

Complications of prolonged food restriction	Prolonged food restriction can increase in the risk of infections, organ complications, need for mechanical ventilation, length of ICU stay and the duration of antibiotic use [1]	Prolonged food restriction can cause fatigue. weakness, disease prolongation, and dysfunction of various organs [16, 17]

Type of food in acute conditions	Numerous studies have shown that the most important factor in the final outcome is the amount of protein intake so that when the amount of protein is the same, calorie reduction does not cause significant change in the final disease outcome [1, 3, 29, 30]	The most important raw food that is considered in food restriction is meat. The amount of meat should be increased in food, if the patient is weak. However, if tolerated by the patient, meat is limited in the early days of the disease and food is prepared using vegetables, grains, and legumes [16, 17]
Increase in food quantity	There is no approved standard for food restriction, but high calorie and protein intake in the first 7 days will worsen prognosis in CIPs. Overeating in the early days will increase the incidence of complications such as hypercapnia, hyperglycemia, uremia, and hypertriglyceridemia [1]	An increase in food in times of crisis exacerbates the disease unless the patient is weak and malnourished. Although it is necessary to reduce the consistency of food, even in weak patients [16, 17]

Changing the consistency of food	In conditions such as acute infections, gastrointestinal disorders, inability to chew, and after surgery, it is recommended to use clear liquids, and liquid, soft and semisolid diets [33, 34]	If the patient isn't weak or malnourished, the patient's food should be more diluted and softer than regular diet. It's similar to clear liquid, liquid, soft, and semisolid diets as described in Table 1 [16, 17]

Preferred route of feeding	If tolerated, intestinal nutrition is the preferred route to nutritionally support CIPs [2, 4, 5]	In the past, intestinal feeding was restricted to intestinal nutrition, which was mainly oral and, in some cases, rectal. Accordingly, the focus was on changing the consistency of food. For example, in patients who were unable to eat, clear liquids, called *Vajoor*, were gently poured into the patient's mouth from the corner of the mouth with special containers [20]

Carbohydrate intake	Due to increased sympathetic activity in the acute phase, insulin resistance and hyperglycemia, it has been recommended to reduce the consumption of carbohydrates [1]	In the absence of fatigue and weakness, vegetables and fruits as carbohydrate sources are a significant part of the recommended foods for patients in the early days of crisis, which are consumed in the form juice, pureed and soup [17]

Protein intake	Food reduction is effective when sufficient amount of protein provided for the patient [1]. Perhaps the importance of proteins is due to the presence of essential amino acids such as leucine, arginine, and glutamine, which play a special role in strengthening the immune system [2, 35, 36]	In case of fatigue, weakness and malnutrition, the most important food component is meat, which should be added to the patient's food in sufficient quantity [16, 17]

Fat intake	The consumption of lipids increases in the acute phase. Because fats produce more energy, they reduce the patient's need for large amounts of food, and of course, they do not increase blood sugar too much and produce less carbon dioxide. Omega-3 fats, unlike omega-6, also help stop the inflammation process. Unlike glucose, fats are well consumed in the acute phase [1]	Fats are used in the patient's food for the following reasons: modifying the food and making it pleasant, softening dry and hard foods, modifying the spiciness of the food, facilitating urination and defecation, facilitating the patient's sleep [37]. In respiratory diseases , special attention has been paid to increase the share of fats in food [17]
